# Intravenous Administration of Adipose-Derived Stem Cell Protein Extracts Improves Neurological Deficits in a Rat Model of Stroke

**DOI:** 10.1155/2017/2153629

**Published:** 2017-02-07

**Authors:** Kai Zhao, Rui Li, Changcong Gu, Long Liu, Yulong Jia, Xize Guo, Wanping Zhang, Chunying Pei, Linlu Tian, Bo Li, Jianrong Jia, Huakun Cheng, Hongwei Xu, Lixian Li

**Affiliations:** ^1^Department of Neurosurgery, The First Affiliate Hospital of Harbin Medical University, Harbin, Heilongjiang 150001, China; ^2^Neuroinflammation Unit, Montreal Neurological Institute, McGill University, Montreal, QC, Canada H3A 2B4; ^3^Department of Immunology, Harbin Medical University, Harbin, Heilongjiang 150086, China; ^4^Department of Neurosurgery, Heilongjiang Provincial Hospital, Harbin, Heilongjiang 150001, China

## Abstract

Treatment of adipose-derived stem cell (ADSC) substantially improves the neurological deficits during stroke by reducing neuronal injury, limiting proinflammatory immune responses, and promoting neuronal repair, which makes ADSC-based therapy an attractive approach for treating stroke. However, the potential risk of tumorigenicity and low survival rate of the implanted cells limit the clinical use of ADSC. Cell-free extracts from ADSC (ADSC-E) may be a feasible approach that could overcome these limitations. Here, we aim to explore the potential usage of ADSC-E in treating rat transient middle cerebral artery occlusion (tMCAO). We demonstrated that intravenous (IV) injection of ADSC-E remarkably reduces the ischemic lesion and number of apoptotic neurons as compared to other control groups. Although ADSC and ADSC-E treatment results in a similar degree of a long-term clinical beneficial outcome, the dynamics between two ADSC-based therapies are different. While the injection of ADSC leads to a relatively mild but prolonged therapeutic effect, the administration of ADSC-E results in a fast and pronounced clinical improvement which was associated with a unique change in the molecular signature suggesting that potential mechanisms underlying different therapeutic approach may be different. Together these data provide translational evidence for using protein extracts from ADSC for treating stroke.

## 1. Introduction

Worldwide, stroke is one of the main causes of death and disability in adult and has become a serious public health problem [1]. Current therapeutic approaches focus on removing the blockage of the blood vessel by thrombolysis (e.g., tPA) and surgery [2, 3]. However, these treatments usually have small time window and have no or limiting effect on neural repair after the injury and, therefore, can only benefit 10% of the patients [2]. There is a call for new therapies that can both limit ischemic brain lesion and promote neural repair.

Recently, several studies using the rodent model of stroke have demonstrated that the administration of mesenchymal stem cells (MSC) can substantially improve the neurological deficits [4]. The potential mechanisms for the therapeutic effect of MSC on stroke may involve secreting neurotrophic factors that promote neural cell survival and growth [5–7], enhancing neurogenesis [6], angiogenesis [6, 8], and modulating neuroinflammatory responses [6, 9]. In addition to these beneficial effects, MSC do not have issues, such as tissue-rejection, as seen in other cell-transplantation-based therapies, which further makes MSC-based therapy an attractive approach for treating stroke. MSC can be isolated from many tissues such as bone-marrow, umbilical cord, and adipose tissue [4, 10]. Compared with other sources, adipose tissue derived MSC (ADSC) have an advantage of being abundant and easy to obtain. ADSC can achieve comparable functional improvement after stroke as compared to other tissue derived MSC [4, 11]. However, the potential risk of tumorigenicity and low survival rate of the implanted cells potentially limit the clinical use of ADSC. ADSC-based cell-free approaches such as using conditioned medium or protein extracts (ADSC-E) may be a feasible approach that could overcome these limitations. Indeed, the therapeutic effect of ADSC-E has been shown in several disease contexts [7, 12–15]. Im et al. showed that ADSC-E can slow down the progression of Huntington's disease by upregulating the expression of p-CREB and PGC-1*α*, two important disease-modifying molecules [13]. Furthermore, administration of human ADSC-E before epilepticus has been shown to lead to the reduction of BBB leakage and earlier attenuation of seizure spike activities after treatment with diazepam [12]. Emerging evidence has suggested that the intraperitoneal administration of ADSC-E decreased ischemic lesion [7]; however, the optimal therapeutic regimen such as the delivery routes and molecular mechanisms still require further study.

The delivery route is important for every cell-based therapy [16, 17]. In some cases, the different delivery routes may result in a completely different clinical outcome. In other cases, although different delivery routes may lead to similar clinical improvement, the underlying mechanisms may be completely different. It has been shown by several studies that either intravenous (IV) or intracerebral (IC) injection of MSC can enhance functional recovery after stroke [1, 7, 10, 18]. However, there is no direct evidence comparing the impact of different routes of administration on the therapeutic effect of MSC.

In this study, we first explored the potential impact of different routes to deliver ADSC-E on stroke. We demonstrated that IV injection of ADSC-E significantly improves the clinical symptom as compared to other control groups. Furthermore, we showed that although ADSC and ADSC-E treatment results in a similar degree of a long-term clinical beneficial outcome, the kinetics of these two ADSC-based therapies are quite different. Such differences between ADSC and ADSC-E are associated with unique molecular signatures. Together these data provide translational evidence for ADSC-E therapy in stroke.

## 2. Materials and Methods

### 2.1. Isolation of Adipose-Derived Mesenchymal Stem Cells (ADSC)

Adipose tissue surrounding the enterocoelia was carefully dissected. The extracted tissue was washed with sterile PBS three times. Next, the tissue was enzymatically digested with an equal volume of 0.2% type I collagenase (Sigma-Aldrich) at 37°C for 60 minutes under shaking conditions. Collagenase activity was neutralized with an equal volume of low glucose Dulbecco's modified Eagle's medium (L-DMEM) containing 10% fetal bovine serum (FBS). The filtered cells were centrifuged at 2000 rpm/min for 10 min to isolate the stromal vascular fraction (SVF). The cell pellet was filtered through a 70 mm pore-size filter. Cells were then cultured in L-DMEM, 1% penicillin/streptomycin, and 10% fetal bovine serum. Culture medium was changed every 24 hrs for 3 days.

### 2.2. Flow Cytometry (FACS) Surface Staining

MSC were validated by flow cytometry. Briefly, ADSC were detached and then washed once with FACS buffer (PBS + 0.5% BSA + 0.1% sodium azide). The cells were incubated with surface antibodies on ice for 20 min. After two times wash, cells were acquired by flow cytometry (Accuri C6, BD). The fluorescence conjugated antibodies for FACS include CD31-PE (Cat#555027, BD), CD44-FITC (203906, Biolegend), CD45-FITC (202205, Biolegend), and CD90-APC (202507, Biolegend). Matched isotype controls are as follows: FITC Mouse IgG2a, *κ* (400207, Biolegend); Alexa Fluor® 647 Mouse IgG1, *κ* (400130, Biolegend); and PE Mouse IgG1, *κ* (550617, BD).

### 2.3. Adipogenic Differentiation of ADSC In Vitro

*Adipogenic Differentiation and Identification of ADSC*. Cells were expanded in 35 mm culture plates until passage 3 or 4 and then were induced for 14 days in an adipogenic induction medium (L-DMEM supplemented with 10% FBS, 0.5 mM isobutylmethylxanthine (IBMX) (Sigma-Aldrich), 1 mM dexamethasone, 10 mM insulin, 200 mM indomethacin, and 1% antibiotic/antimycotic solution). The culture medium was changed every 3 days. At Day 14, cells were fixed in 4% formalin and stained with 2% fresh Oil red-O solution (Sigma-Aldrich) to detect lipid droplets in the induced cells.

### 2.4. Osteogenic Differentiation of ADSC In Vitro

ADSCs at passage 3 (P3) were incubated at 1 × 10^4^ cells/cm^2^ in a 35 mm culture plate. The induction medium consisting of L-DMEM, 10% FBS, 0.1 *μ*M dexamethasone, 10 mM *β*-glycerophosphate, 0.05 mM ascorbate-2- phosphate, and 1% antibiotic/antimycotic solution was used to induce osteogenic differentiation in vitro. The culture medium was changed every 3 days. At Day 21, cells were fixed with 4% paraformaldehyde and then gently washed with deionized water and incubated for 1 h in a 5% (wt/vol) silver nitrate solution under UV light; the cells were washed 3 times, with neutralization of 5% sodium thiosulfate. Finally, the cells were observed and photographs were taken under an inverted microscope.

### 2.5. ADSC Extract (ADSC-E) Preparation

The ADSC-E were freshly prepared before injection. Briefly, 1 × 10^6^ ADSC were harvested and washed twice with PBS. Cells were then suspended in lysis buffer (1 mM DTT, 1 mM EDTA, protease inhibitor cocktail P8340, 0.1% DEPC in PBS) and lysed within a syringe tube with a plugged end by pushing and pulling the syringe piston several times. Then put the tubes into liquid nitrogen and water at 37°C about several times. Cell lysates were then centrifuged at 14,000*g* for 15 min. The final products (around 600 ug) were later filtered through 0.45 uM filter.

### 2.6. Animals and Grouping

Adult Sprague-Dawley rats were used, with an average body weight range of 250–320 gram and average age range of 4–6 weeks. The animals were housed with free access to food and water at a room temperature. All experimental procedures involved were performed based on protocols that are reviewed and approved by the Institutional Animal Care and Use Committee at Harbin Medical University. The animals were randomly assigned to one of five experimental groups: (1) the sham-operated group, which underwent surgery without infarct; (2) the control group, which underwent surgery with transient middle cerebral artery occlusion (tMCAO) and received PBS infusion in the femoral vein, intraperitoneal or intracerebral (different delivery routes alone without ADSC-E had no effect on cerebral ischemia (data not shown)); (3) the ADSC group, which underwent tMCAO surgery and received ADSC infusion in the femoral vein; (4) the ADSC-E group, which underwent tMCAO surgery and received an ADSC-E infusion in the femoral vein, intraperitoneal or intracerebral.

### 2.7. Transient Middle Cerebral Artery Occlusion (tMCAO) Model

Anesthesia was induced by intraperitoneal injection of a solution of 10% chloral hydrate at a dose of 350 mg/kg, maintaining their rectal temperature at 37°C. The left common carotid artery (CCA), external carotid artery (ECA), and internal carotid artery (ICA) of each rat were exposed via a midline neck incision. The tip of a 4-0 nylon monofilament was rounded by heating over a flame and coated with silicone. To occlude the origin of the middle cerebral artery (MCA), the monofilament was moved carefully into the ICA lumen via the ECA lumen until a slight resistance was felt. After 2 h, the suture was slowly withdrawn to allow reperfusion. The rats in the sham group received all the surgical procedures in the absence of a suture. Neurological function was evaluated by mNSS scaling system and Roger's functional scale as described before [19, 20] by two researchers who were blinded to the experimental groups.

### 2.8. Cell Administration

ADSCs cultured at passage 3 to 5 were collected by trypsin (0.25%) digestion. Thus, intravenous injections of ADSC and ADSC-E, heated ADSC-E, DNA, and RNA derived from equal amounts of ADSC (around 1 × 10^6^) in 650 *μ*l PBS were administered for 5 min through the femoral vein after common carotid artery reperfusion, and intracerebral injections of ADSC-E in 15 *μ*l PBS were administered through a Hamilton syringe in a stereotactic apparatus (AP-1.0 mm; ML-1.5 mm; DV-3.5 to 4.5 mm) immediately after CCA reperfusion, the process for about 10 minutes.

### 2.9. Neurological Function Evaluation

Neurological function was evaluated by modified neurological severity scores (mNSS) scaling system and Roger's functional scale as described before by two researchers who were blinded to the experimental groups. The mNSS was defined as follows: raising rat by the tail (3); placing rat on the floor (normal = 0; maximum = 3); sensory test (2); beam balance tests (normal = 0; maximum = 6); reflexes absent and abnormal movements (4), and maximum points (18). In all, 1 to 6 indicates mild injury; 7 to 12, moderate injury; and 13 to 18, severe injury. A variant of Roger's functional scale was used to assign scores as follows: 0, no functional deficit; 1, failure to extend forepaw fully; 2, decreased grip of forelimb while tail is gently pulled; 3, spontaneous movement in all directions, contralateral circling only if pulled by the tail; 4, circling; 5, walking only when stimulated; 6, unresponsive to stimulation with a depressed level of consciousness; and 7, dead.

### 2.10. 2,3,5-Triphenyltetrazolium Chloride (TTC) Staining

The brain tissues were sliced into seven 2 mm thick coronal sections and stained with 1% TTC solution at 37°C for 15 min. Each section was then washed with PBS and fixed in 4% paraformaldehyde. The infarction volume was presented as a percentage of the volume of the brain. Image J software was used to analyze the percentage of TTC-stained tissue.

### 2.11. TUNEL Staining

Cell death was detected by terminal deoxynucleotidyl transferase (TUNEL) staining following manufacturer's protocol. Briefly, the section was first fixed using 4% paraformaldehyde and then incubated in the permeabilization buffer (0.1% Triton X-100 in 0.1% sodium citrate) on ice for 2 min. The section was then incubated in TUNEL reaction mixture (enzyme solution and label solution) for 60 min at 37°C. After that, the reaction was terminated by adding 1X PBS solution for 15 min. Nuclei were then counterstained with DAPI for 5 min.

### 2.12. Real-Time Quantitative PCR Analysis

The expression levels of IL-6 (forward primer: 5′-CAGGGAGATCTTGGAAATGAG-3′; reverse primer: 5′-GTTGTTCTTCACAAACTCC-3′), TNF*α* (forward primer: 5′-GATCGGTCCCAACAAGGAGG-3′; reverse primer: 5′-GCTGGTACCACCAGTTGGTTG-3′), IFN*γ* (forward primer: 5′-AGGAAAGAGCCTCCTCTTGG-3′; reverse primer: 5′-TGGGTTGTTCACCTCGAACT-3′), IL-10 (forward primer: 5′-TAAGGGTTACCTTGGGTTGCCAAGCC-3′; reverse primer: 5′-AGGGGAGAAATCGATGACAGCGCC-3′), BDNF (forward primer: 5′-CGACGTCCCTGGCTGACACTTTT-3′; reverse primer: 5′-AGTAAGGGCCCGAACATACGATTGG-3′), NGF (forward primer: 5′-ACCTCTTCGGACACTCTGG-3′; reverse primer: 5′-CGTGGCTGTGGTCTTATCTC-3′), CNTF (forward primer: 5′-TCCAAGAGAACCTCCAGGCTTA-3′; reverse primer: 5′-GCTGGTAGGCAAAGGCAGAA-3′), IGF-1 (forward primer: 5′-GACATGCCCAAGACCCAGAAGGA-3′; reverse primer: 5′-CGGTGGCATGTCACTCTTCACTC-3′), and *β*-actin (forward primer: 5′-ACGTTGACAT CCGTAAAGAC-3′; reverse primer: 5′-GAAGGTGGACAGTGAGGC-3′) were measured by real-time PCR (RT-PCR). Total RNA of each sample was extracted from the tissue of the infarcted border zones using Trizol (Invitrogen) according to the manufacture's protocol, and we conducted RT-PCR using the First-Strand cDNA Synthesis Kit (Roche). Quantitative real-time RT-PCR was performed using SYBR-Green (Roche).

### 2.13. Statistic

GraphPad Prism 6 was used to perform all the statistical analysis. One-way ANOVA was used for statistical comparisons among more than two groups. All statistical tests have been indicated in the figure legends. *p* values of 0.05 or less were considered significant.

## 3. Results

### 3.1. ADSC Characterization

**Figure 1:**
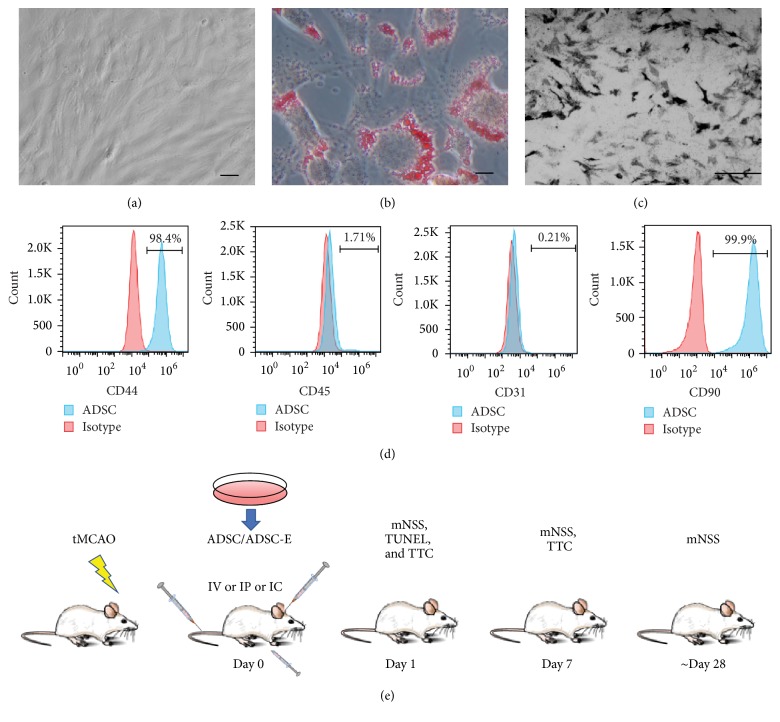
ADSCs characterization and ADSC treatment regime. ADSC were isolated from adipose tissue surrounding the enterocoelia. As ADSC approached confluence, they showed a more spindle-shaped, fibroblastic morphology. Scale bars: 100 um (a). (b) ADSC were cultured in the adipogenic induction medium for 14 days. Oil red was used to validate the induction. The intracellular lipid droplets were showed as positive red loci. Scale bars: 100 um (c). ADSC were cultured in the osteogenic induction medium for 21 days. Von Kossa staining showed that a calcified extracellular matrix of induced ADSC was detected. Scale bars: 500 um (d). Flow cytometry analysis was used to measure the surface marker of ADSCs. ADSCs were negative for leukocyte markers, CD45 (1.71%) and CD31 (0.21%), and positive for mesenchymal stem cell markers, CD44 (98.4%) and CD90 (99.9%). (d). ADSC/ADSC-E were administrated through IV or IC or IP 1 hour after tMCAO (e).

ADSC were isolated from adipose tissue surrounding the enterocoelia. The primary culture of ADSC appeared as a monolayer of large, flat cells. As the cells approached confluence, they showed a more spindle-shaped, fibroblastic morphology (Figure 1(a)). To test adipogenic differentiation of ADSC, ADSC were cultured in the adipogenic induction medium for 14 days. Oil red was used to validate the induction. As shown in Figure 1(b), the intracellular lipid droplets were showed as positive red loci (Figure 1(b)). To test osteogenic differentiation in vitro, ADSC were cultured in the osteogenic induction medium for 21 days. Von Kossa staining showed that a calcified extracellular matrix of induced ADSC was detected, which confirmed ADSC could differentiate into osteogenic cells (Figure 1(c)). Flow cytometry analysis of ADSC at passages 3-4 demonstrated that the cells were negative for leukocyte and endotheliocyte markers, CD45 (1.71%) and CD31 (0.21%), and positive for mesenchymal stem cell markers, CD44 (98.4%) and CD90 (99.9%) (Figure 1(d)).

### 3.2. The Impact of Delivery Routes on the Therapeutic Effect of Protein Extracts from ADSC during Stroke

**Figure 2:**
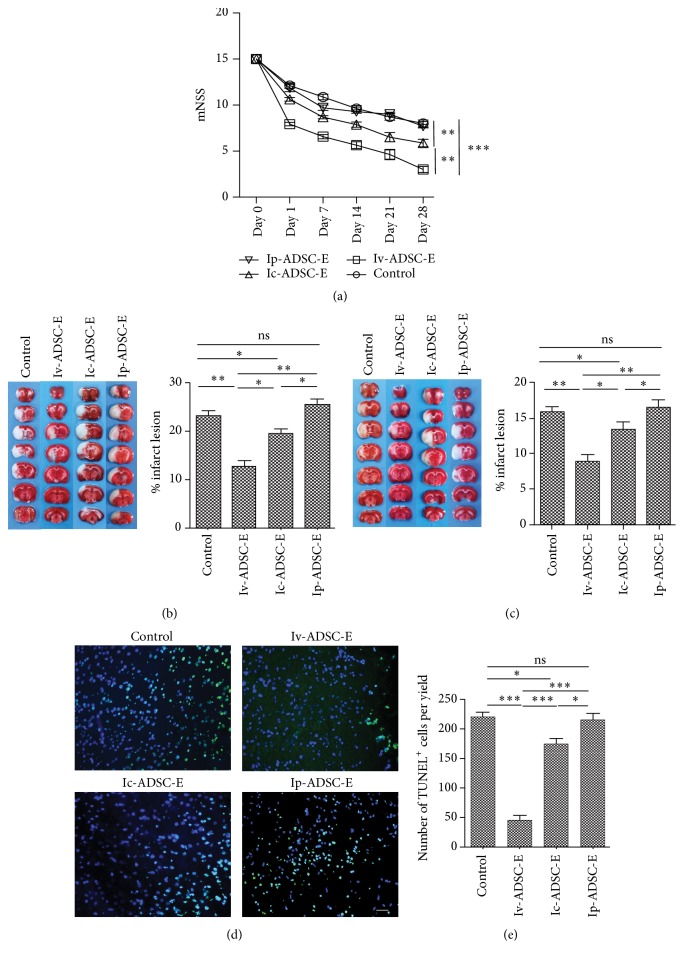
The impact of delivery routes on the therapeutic effect of ADSC-E during stroke. tMCAO was induced using SD rat. Protein extracts from ADSCs (ADSC-E) were injected either through IV, IC, or IP after the operation. (a) The neurological deficits were graded based on mNSS scaling system (*n* = 8/group). (b, c) TTC staining was used to quantify the volume of infarct area at either Day 1 (*n* = 6/group) (b) or Day 7 (*n* = 6/group) (c). (d, e) TUNEL staining was used to measure apoptotic neurons. Scale bars: 100 um (e). Five sections from each mouse were selected (*n* = 3/group). For each section, TUNEL positive neural cells were counted from 3 separated yields. ANOVA was used to analyze the data. ^*∗*^*p* < 0.05, ^*∗∗*^*p* < 0.01, and ^*∗∗∗*^*p* < 0.001.

Little is known how different delivery routes may influence the therapeutic effect of ADSC-E. To test how different delivery routes may impact the therapeutic effect of ADSC-E on stroke, we induced tMCAO on SD Rat. Protein extracts from prevalidated ADSC were injected either intravenous (IV) or intracerebral (IC) or intraperitoneal (IP) to the tMCAO rats. Neurological deficits were graded based on modified neurological severity scores (mNSS) scale system from Day 0 to Day 28 (Figure 1(e)). TTC staining was used to quantify the volume of infarct lesion at Day 1 and Day 7 (Figure 1(e)). Inconsistent with previous work [7], we showed that injection of ADSC-E through IP did not improve either neurological deficits or ischemic lesion (Figures 2(a)–2(c)). Compared to IC, IV injection of ADSC-E showed much better improvement of the neurological deficits of both mNSS scaling system (Figure 2(a)) and Roger's functional scale (Supplementary 1A in Supplementary Material available online at https://doi.org/10.1155/2017/2153629 ). In keeping with the clinical evaluation, the volume of infarct region was also reduced in the IV group and to a lesser extent in the IC group at both Day 1 and Day 7 (Figures 2(b) and 2(c)). Furthermore, the number of apoptotic neuron in the penumbra area of the ischemic lesion is also remarkably decreased in the IV group (Figures 2(d) and 2(e)). In addition, we noticed that the operation procedure of intracerebral injection significantly increases the rate of death (data not shown). Taken together, compared to other delivery routes, administration of ADSC-E through IV is relatively safe and highly effective way to deliver ADSC.

### 3.3. The Impact of Different Components of ADSC during Stroke

Previous study has found Neuro2a cells that are treated with the ADSC-E increased cell viability and reduced cytotoxicity [7]. However, most of the cells treated with heat-treated ADSC-E, DNA, RNA, or lipids derived from ADSC did not show similar protection. But this has never been tested in vivo. To test which kind of the cellular components of ADSC have the most robust therapeutic effect on stroke, we intravenously inject ADSC-E, heat-treated ADSC-E, DNA, RNA, or vehicle after tMCAO. We found that, in the ADSC-E group, on the first Day after treatment, the neurological function was restored (Supplementary 2A and 2B), and the infarct size was significantly reduced at Day 1 (Supplementary 2C). However, no differences were observed in the other groups, suggesting that the protein component of ADSC-E was responsible for the therapeutic effect of ADSC on stroke.

### 3.4. Different Therapeutic Dynamics between ADSC and ADSC-E on Stroke

**Figure 3:**
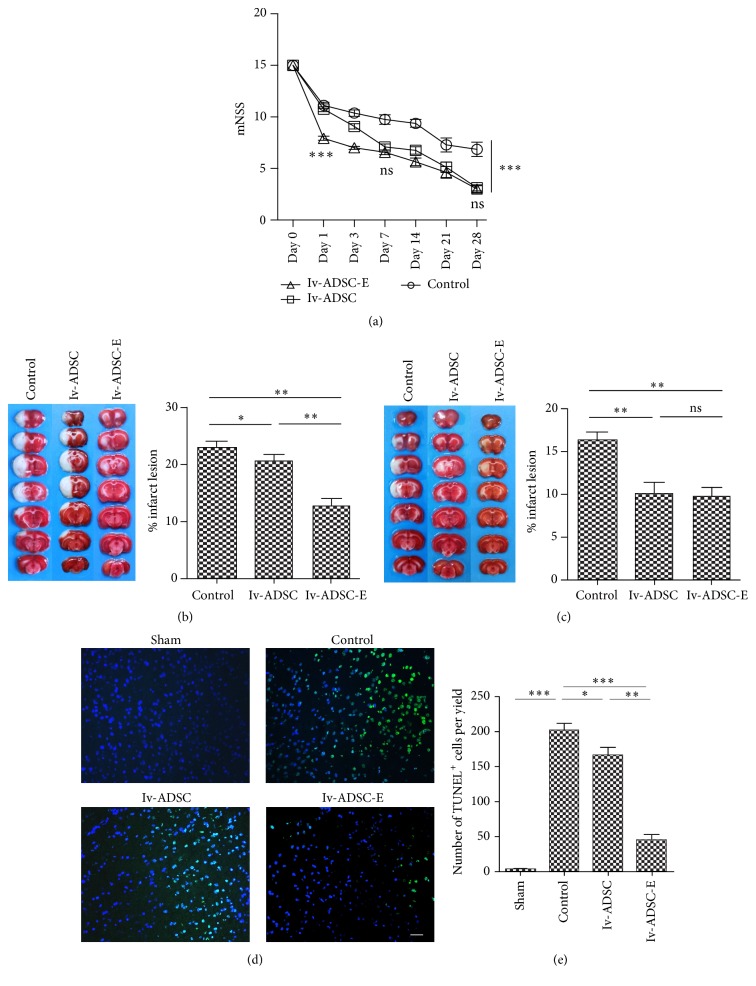
Different therapeutic characteristics between ADSC and ADSC-E on stroke. tMCAO was induced using SD rat. Either ADSC or ADSC-E was injected through IV. (a) The neurological deficits were graded based on mNSS scaling system (*n* = 8/group). (b, c) TTC staining was used to quantify the volume of infarct area at either Day 1 (*n* = 6/group) (b) or Day 7 (*n* = 6/group) (c). (d, e) TUNEL staining was used to measure apoptotic neurons. Scale bars: 100 um (e). Five sections from each rat were selected (*n* = 3/group). For each section, TUNEL positive neural cells were counted from 3 separated yields. ANOVA was used to analyze the data. ^*∗*^*p* < 0.05, ^*∗∗*^*p* < 0.01, and ^*∗∗∗*^*p* < 0.001.

Next, we would like to know the potential differences between adipose-derived stem cell and protein extracts from ADSC in stroke therapy. Either ADSC (1 × 10^6^) or ADSC-E (generated from 1 × 10^6^) were injected through IV to the rats that received tMCAO. We found that although both ADSC and ADSC-E can significantly improve the neurological deficits both mNSS scaling system (Figure 3(a)) and Roger's functional scale (Supplementary 1B) and reduce the infarct lesion (Figure 3(c)), the dynamics of these two treatments are different. The administration of ADSC-E results in a much faster neurological improvement while the injection of ADSC leads to a relatively modest but prolonged relief of the clinical symptoms (Figure 3(a)). Consistent with the clinical evaluation, TTC staining and TUNEL staining further supported that the treatment of ADSC-E results in less neuronal damage at earlier time points (Days 1~3) (Figures 3(b) and 3(d)). These together suggested that the therapeutic dynamics between ADSC and ADSC-E on stroke is different.

### 3.5. Neuroprotective Function of ADSC and Immunomodulatory Roles of ADSC-E Are Associated with the Differences of Therapeutic Dynamics between ADSC and ADSC-E

**Figure 4:**
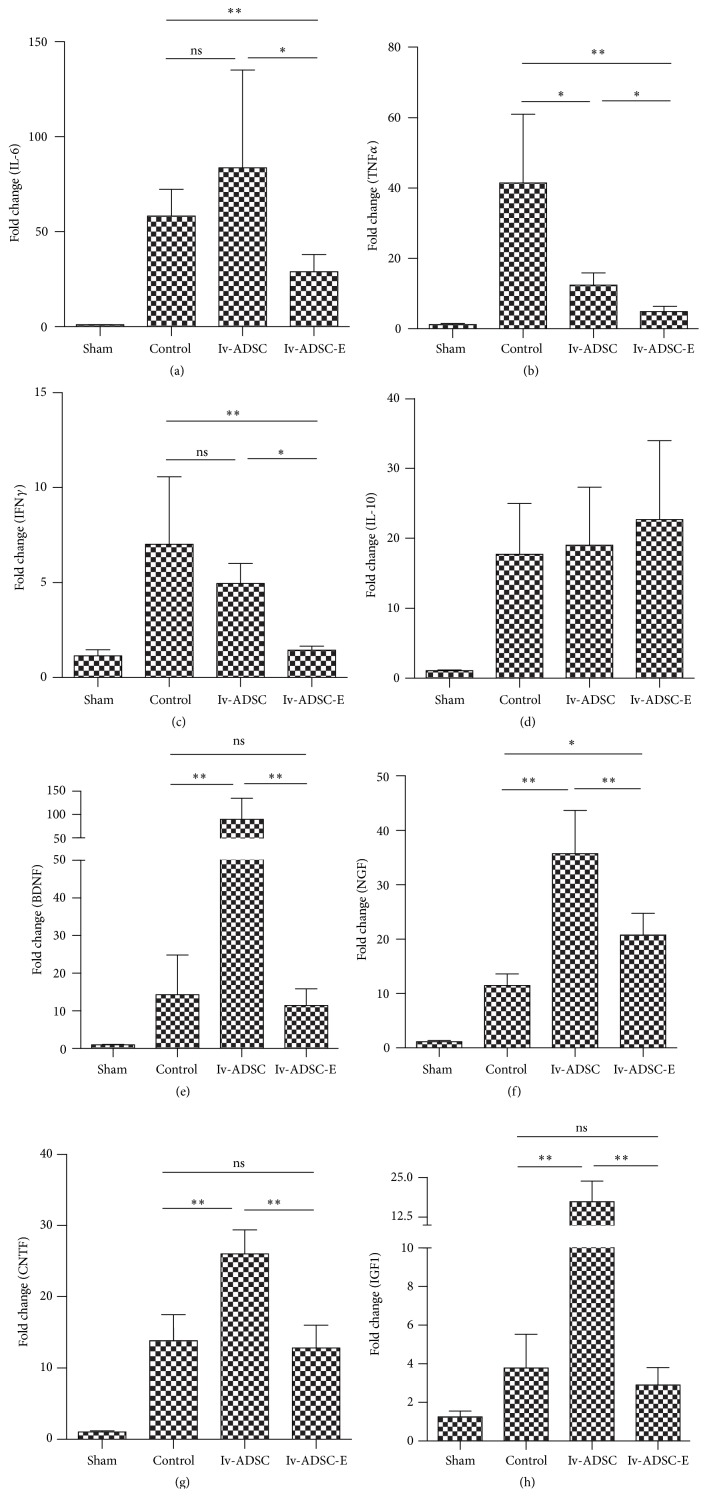
Different molecular signatures between ADSC and ADSC-E on stroke therapies. tMCAO was induced using SD rat. Either ADSC or ADSC-E was injected through IV. mRNA was extracted from the ipsolateral cortex. Cytokines (both pro- and anti-inflammatory) ((a)–(d), mRNA from Day 1) and neurotrophic factors ((e)–(h), mRNA from Day 3) were measured by real-time PCR (*n* = 6/group). ANOVA was used to analyze the data. ^*∗*^*p* < 0.05; ^*∗∗*^*p* < 0.01.

Next, we would like to further explore the potential reason for the difference of therapeutic dynamics between ADSC and ADSC-E. Studies have suggested that “bystander” functions, including upregulation of neurotrophic factors, downregulation of proinflammatory cytokines, and enhancing angiogenesis, are the main contributors to the therapeutic effect of ADSC during the stroke. We asked whether the phenomenon that we observed above may be due to the differential molecular/cellular mechanisms underlying different ADSC-based therapeutic approaches. To test this hypothesis, we measured the expression of proinflammatory cytokines and neurotrophic factors by real-time PCR using mRNA isolated from the ipsolateral cortex of the rat with or without ADSC-based therapies either at Day 1 or Day 3. We noticed that ADSC-E significantly downregulated proinflammatory cytokine (IL-6, TNF*α*, and IFN*γ*) production in the ischemic brain (Figures 4(a)–4(d)), while the injection of ADSC only reduced TNF*α* (Figure 4(b)), which suggested that ADSC-E, at the earlier time point, showed a stronger immunomodulatory effect as compared to ADSC. In contrast, the upregulation of neurotrophic factors (BDNF, NGF, CNTF, and IGF) is much more robust in the ADSC-treated group as compared to the group which received ADSC-E (Figures 4(f)–4(h)) at Day 3, which may explain the prolonged effect of ADSC. Collectively, these data indicated that the different therapeutic dynamics between ADSC and ADSC-E maybe related to their differential modes of action.

## 4. Discussion

Studies have shown that stem cell-based therapies can substantially improve the neurological deficits during stroke [4, 11]. Our current study explored the potential usage of a “cell-free” ADSC-based approach in stroke therapy. We showed that IV injection of the protein extract but not injecting other components of ADSC substantially improves the neurological deficits and rescues neuron from death. In addition, compared to ADSC, ADSC-E treatment results in a much faster clinical improvement, which is associated with its stronger ability to modulate neuroinflammation.

The choice of delivery routes is critically important for cell-based therapy which may directly influence outcomes of the treatment. The ideal delivery route should be relatively safe, cost efficient, and easy to apply to the patients without causing any additional issue. Furthermore, cells administered by this route should result in benefits to clinical outcome measures. The delivering routes utilized for MSC/ADSC-based therapy on stroke include intracerebral (IC), intravenous (IV), and intraperitoneal (IP) [7, 8, 10, 17, 18]. However, no study has directly compared the effectiveness of different delivery routes on ADSC-based therapies during the stroke. In this study, we have demonstrated that the therapeutic effect of ADSC-based therapies on stroke is strongly associated with the delivery routes. In contrast to previously published study, we did not observe any beneficial effect of ADSC-E injected through peritoneal administration. This may be due to the differences of cell number to generate protein extracts. In the current study, we injected protein extract that is isolated from 5 times less ADSC than the previous study [7]. Despite this, we still see a substantial clinical beneficial effect in the IV group, further suggesting that IV is a preferable choice for ADSC-E in stroke therapy.

There are several advantages of using “cell-free” ADSC-based over cell-based ADSC therapy. Firstly, protein extracts are more readily to use and easier to preserve. Secondly, although the therapeutic effect of cell-free approach maybe transient due to the short half-life of the effector protein, “cell-free” approach also eliminated the potential long-term side effect of cell-based approach such as tumorigenesis. In addition, our data suggested that the treatment of ADSC-E results in a faster therapeutic effect in stroke, which is extremely valuable and may open therapeutic windows for other combination therapies.

Although evidence has shown that ADSC can differentiate into neural cells in vitro [21, 22], recent studies indicated that very few ADSC can actually differentiate into neural cells in vivo [23, 24], which may due to the lack of permissive local environment for neural differentiation [4], suggesting that neural cell replacement may not be the primary mechanism underlying the therapeutic effect of ADSC on stroke. The effectiveness of using protein extract or conditional medium from ADSC on stroke further supports this [7, 25]. Immune modulatory function may be important for the therapeutic function of ADSC during stroke [4, 9, 26, 27]. The molecular mechanisms may involve immune regulatory molecules such as TGFbeta [28, 29], IDO [30], and PGE2 [31, 32]. However, little is known about the place where ADSC regulates the immune responses: peripheral lymphoid tissue or local CNS or both. Our data have shown that IV injection of ADSC-E has a stronger impact on limiting ischemic lesion and apoptotic lesion as compared to IC injection of ADSC-E, which may indirectly indicate that the effect of ADSC-E on the peripheral immune responses maybe more important mechanism underlying the therapeutic effect of ADSC-E.

## 5. Conclusion

Taken together, we conclude that IV injection of protein extracts from ADSC showed a faster clinical outcome as compared to ADSC. Different therapeutic dynamics between ADSC and ADSC-E may be due to different modes of action. Our study provides the translational basis for using protein extracts from ADSC in stroke therapies.

## Supplementary Material

Supplementary Figure 1. Measurement of Neurological deficits using Roger's scaling system. (A) tMCAO was induced using SD rat. Protein extract from ADSCs (ADSC-E) was injected either through IV, IC or IP after the operation. The impact of different delivery routes on the therapeutic effect of ADSC-E during stroke were measured by Roger's scaling system (n=8/group). (B) tMCAO was induced using SD rat. Either ADSC or ADSC-E was injected through IV. The therapeutic characteristics between ADSC and ADSC-E on behavior change were measured by Roger's scaling system (n=8/group).Supplementary Figure 2. The impact of different components of ADSC during stroke. tMCAO was induced using SD rat. Different components (ADSC-E, Heated-ADSC-E, RNA and DNA) of ADSC were injected through IV. (A, B). Functional outcome was measured by mNSS Scaling system (n=4/group) (A) and Roger's Scaling system (n=4/group) (B). (C, D). Lesion size was measured using TTC staining at day 1 (n=4/group).
